# Ethyl 5-oxo-4-phenyl-5,6-di­hydro-4*H*-1,3,4-oxadiazine-2-carboxyl­ate

**DOI:** 10.1107/S1600536814011106

**Published:** 2014-05-17

**Authors:** K. Shubakara, Chandra, N. Srikantamurthy, M. Mahendra, K. B. Umesha

**Affiliations:** a Department of Chemistry, Yuvaraja’s College, University of Mysore, Mysore 570 005, India; b Department of Studies in Physics, Manasagangotri, University of Mysore, Mysore 570 006, India

## Abstract

The asymmetric unit of title compound, C_12_H_12_N_2_O_4_, consists of two independent mol­ecules. In each mol­ecule, the oxadiazine ring has a flattened envelope conformation with the methyl­ene C atom as the flap atom, and the eth­oxy­carbonyl unit is in a *syn-periplanar* conformation with respect to the oxadiazine ring as indicated by O—C—C=O torsion angles of 1.9 (4) and 2.5 (4)°. The dihedral angles between the mean plane of the oxadiazine ring and the phenyl ring are 80.07 (13) and 42.98 (14)°. In the crystal, mol­ecules are linked by C—H⋯O hydrogen bonds and stacked in a double-column along the *a-*axis direction.

## Related literature   

For the biological activity of oxadiazine derivatives, see: Barbari *et al.* (2003 ▶); Gsell & Maientisch (1998 ▶). For a related structure, see: Chopra *et al.* (2004 ▶). For puckering parameters, see: Cremer & Pople (1975 ▶).
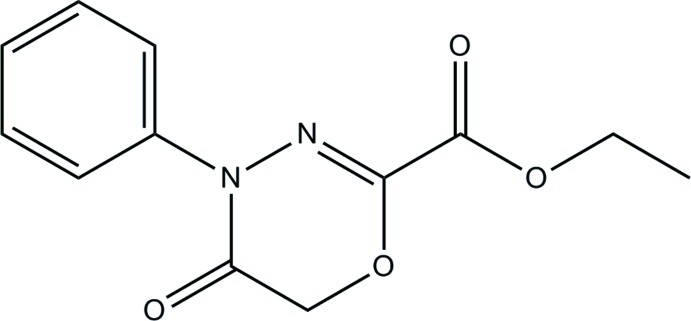


## Experimental   

### 

#### Crystal data  

C_12_H_12_N_2_O_4_
*M* *_r_* = 248.24
Triclinic, 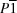

*a* = 9.3499 (7) Å
*b* = 9.3601 (8) Å
*c* = 15.2707 (15) Å
α = 104.007 (8)°
β = 99.366 (7)°
γ = 107.862 (7)°
*V* = 1192.9 (2) Å^3^
*Z* = 4
Mo *K*α radiation
μ = 0.11 mm^−1^
*T* = 296 K
0.30 × 0.25 × 0.20 mm

#### Data collection  

Bruker APEXII CCD area-detector diffractometer
9756 measured reflections
4176 independent reflections
2173 reflections with *I* > 2σ(*I*)
*R*_int_ = 0.034

#### Refinement  

*R*[*F* ^2^ > 2σ(*F* ^2^)] = 0.054
*wR*(*F* ^2^) = 0.177
*S* = 1.03
4176 reflections
327 parameters
H-atom parameters constrained
Δρ_max_ = 0.20 e Å^−3^
Δρ_min_ = −0.20 e Å^−3^

### 

Data collection: *APEX2* (Bruker, 2009 ▶); cell refinement: *SAINT* (Bruker, 2009 ▶); data reduction: *SAINT*; program(s) used to solve structure: *SHELXS97* (Sheldrick, 2008 ▶); program(s) used to refine structure: *SHELXL97* (Sheldrick, 2008 ▶); molecular graphics: *PLATON* (Spek, 2009 ▶); software used to prepare material for publication: *SHELXL97*.

## Supplementary Material

Crystal structure: contains datablock(s) global, I. DOI: 10.1107/S1600536814011106/is5362sup1.cif

Structure factors: contains datablock(s) I. DOI: 10.1107/S1600536814011106/is5362Isup2.hkl

Supporting information file. DOI: 10.1107/S1600536814011106/is5362Isup3.cml

CCDC reference: 1003020

Additional supporting information:  crystallographic information; 3D view; checkCIF report

## Figures and Tables

**Table 1:** Hydrogen-bond geometry (Å, °)

*D*—H⋯*A*	*D*—H	H⋯*A*	*D*⋯*A*	*D*—H⋯*A*
C2*A*—H2*A*2⋯O1*A* ^i^	0.97	2.55	3.150 (3)	120
C14*B*—H14*A*⋯O3*A* ^ii^	0.97	2.59	3.418 (4)	143
C14*B*—H14*B*⋯O5*B* ^ii^	0.97	2.57	3.163 (3)	120

Symmetry codes: (i) 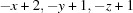; (ii) 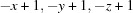.

**Fig. 1.**
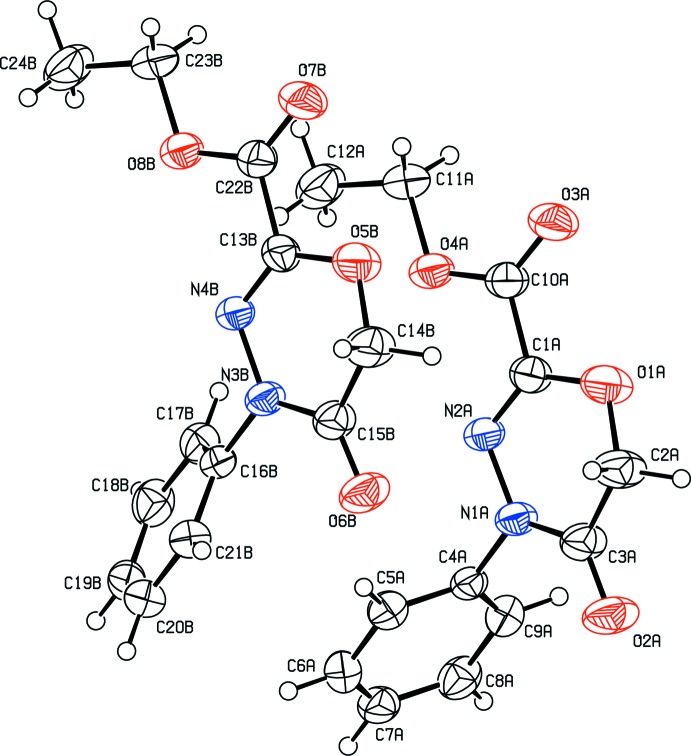
Perspective diagram of the title compound with 50% probability displacement ellipsoids.

**Fig. 2.**
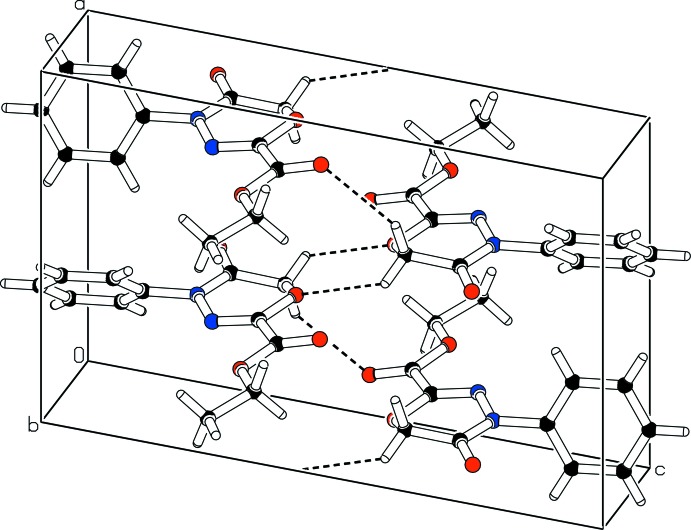
Packing diagram of the title compound viewed down the b axis.

## supplementary crystallographic information

### 1. Comment

Heterocyclic compounds containing nitrogen and oxygen atoms are of great synthetic interest due to their versatile biological significance. Oxadiazine derivatives are one among those heterocyclic compounds exhibiting various biological activities, for instance 1,2,5-oxadiazine-3,6-diones are potent antiviral agents (Barbari *et al.*, 2003). Also, as an important type of insecticides, oxadiazine derivatives are highly efficient and of low toxicity (Gsell & Maientisch, 1998). With this background on oxadiazine derivatives, we have synthesized the title compound to study its crystal structure.

The two independent molecules (A and B) of the title compound (Fig. 1) in the asymmetric unit exhibit highly planar conformation, with their maximum deviations on ring planes at N1A and N3B are 0.081 (2) Å and 0.055 (2) Å, respectively. The central oxadiazine moiety adopts a flattened envelope conformation with puckering parameters Q(2) = 0.281 (3) Å, Q(3) = 0.118 (3) Å and φ = 325.4 (6)° (Cremer & Pople, 1975). The bond lengths and angles are generally within normal ranges and are comparable to a related structure (Chopra *et al.*, 2004). In the molecules A and B, the oxadiazine moiety makes a dihedral angle of 80.07 (13) and 42.98 (14)°, with the phenyl rings (C4A–C9A and C16B–C21B), respectively. The ethoxycarbonyl unit is in a *syn-periplanar* conformation with respect to the oxadiazine moiety, as indicated by the torsion angles of 1.9 (4)° (O1A—C1A—C10A—O3A) and 2.5 (4)° (O5B—C13B—C22B—O7B) for A and B, respectively. The crystal structure is stabilized by C—H···O hydrogen bonds and the molecules are stacked in a column along the *a* axis (Fig. 2).

### 2. Experimental

Ethyl 5-oxo-4-phenyl-5,6-dihydro-4*H*-1,3,4-oxadiazine-2-carboxylate were obtained from ethyl 2-oxo-2-(2-phenylhydrazinyl) acetate by one pot condensation-cyclization reaction with chloroacetylchloride using potassium carbonate in dry acetone as a solvent. Compounds were purified by column chromatography using petroleum ether and acetone in (2:8) as eluent.

### 3. Refinement

H atoms were placed at idealized positions and allowed to ride on their parent atoms with C—H distances in the range of 0.93 to 0.97 Å, and with *U*_iso_(H) = 1.2 or 1.5*U*_eq_(C).

### Crystal data

**Table:**

C_12_H_12_N_2_O_4_	*Z* = 4
*M**_r_* = 248.24	*F*(000) = 520
Triclinic, *P*1	*D*_x_ = 1.382 Mg m^−^^3^
Hall symbol: -P 1	Mo *K*α radiation, λ = 0.71073 Å
*a* = 9.3499 (7) Å	Cell parameters from 4176 reflections
*b* = 9.3601 (8) Å	θ = 2.4–25.0°
*c* = 15.2707 (15) Å	µ = 0.11 mm^−^^1^
α = 104.007 (8)°	*T* = 296 K
β = 99.366 (7)°	Block, colourless
γ = 107.862 (7)°	0.30 × 0.25 × 0.20 mm
*V* = 1192.9 (2) Å^3^	

### Data collection

**Table:**

Bruker APEXII CCD area-detector diffractometer	*R*_int_ = 0.034
ω and φ scans	θ_max_ = 25.0°, θ_min_ = 2.4°
9756 measured reflections	*h* = −11→11
4176 independent reflections	*k* = −11→11
2173 reflections with *I* > 2σ(*I*)	*l* = −18→18

### Refinement

**Table:**

Refinement on *F*^2^	Primary atom site location: structure-invariant direct methods
Least-squares matrix: full	Secondary atom site location: difference Fourier map
*R*[*F*^2^ > 2σ(*F*^2^)] = 0.054	Hydrogen site location: inferred from neighbouring sites
*wR*(*F*^2^) = 0.177	H-atom parameters constrained
*S* = 1.03	*w* = 1/[σ^2^(*F*_o_^2^) + (0.0687*P*)^2^ + 0.0133*P*] where *P* = (*F*_o_^2^ + 2*F*_c_^2^)/3
4176 reflections	(Δ/σ)_max_ < 0.001
327 parameters	Δρ_max_ = 0.20 e Å^−^^3^
0 restraints	Δρ_min_ = −0.20 e Å^−^^3^

### Special details

**Table:**

Geometry. Bond distances, angles *etc*. have been calculated using the rounded fractional coordinates. All su's are estimated from the variances of the (full) variance-covariance matrix. The cell e.s.d.'s are taken into account in the estimation of distances, angles and torsion angles
Refinement. Refinement on *F*^2^ for ALL reflections except those flagged by the user for potential systematic errors. Weighted *R*-factors *wR* and all goodnesses of fit *S* are based on *F*^2^, conventional *R*-factors *R* are based on *F*, with *F* set to zero for negative *F*^2^. The observed criterion of *F*^2^ > σ(*F*^2^) is used only for calculating -*R*-factor-obs *etc*. and is not relevant to the choice of reflections for refinement. *R*-factors based on *F*^2^ are statistically about twice as large as those based on *F*, and *R*-factors based on ALL data will be even larger.

### Fractional atomic coordinates and isotropic or equivalent isotropic displacement parameters (Å^2^)

**Table:**

	*x*	*y*	*z*	*U*_iso_*/*U*_eq_	
O1A	0.9018 (3)	0.5053 (2)	0.41230 (12)	0.0818 (9)	
O2A	0.9228 (3)	0.1798 (2)	0.24645 (13)	0.0852 (9)	
O3A	0.8395 (2)	0.7645 (2)	0.47867 (12)	0.0751 (8)	
O4A	0.7043 (2)	0.7358 (2)	0.33599 (11)	0.0627 (7)	
N1A	0.8228 (2)	0.3648 (2)	0.22576 (13)	0.0497 (7)	
N2A	0.7753 (2)	0.4882 (2)	0.26292 (14)	0.0496 (8)	
C1A	0.8179 (3)	0.5490 (3)	0.35128 (17)	0.0523 (9)	
C2A	0.8926 (4)	0.3456 (3)	0.38006 (18)	0.0762 (13)	
C3A	0.8834 (3)	0.2883 (3)	0.27857 (18)	0.0590 (10)	
C4A	0.7924 (3)	0.3134 (3)	0.12527 (16)	0.0431 (8)	
C5A	0.6417 (3)	0.2277 (3)	0.07184 (16)	0.0523 (9)	
C6A	0.6136 (3)	0.1792 (3)	−0.02421 (17)	0.0590 (10)	
C7A	0.7342 (3)	0.2172 (3)	−0.06527 (17)	0.0583 (10)	
C8A	0.8838 (3)	0.3026 (3)	−0.01176 (17)	0.0586 (10)	
C9A	0.9132 (3)	0.3525 (3)	0.08430 (17)	0.0540 (9)	
C10A	0.7883 (3)	0.6949 (3)	0.39674 (18)	0.0538 (9)	
C11A	0.6807 (3)	0.8841 (3)	0.37234 (19)	0.0649 (11)	
C12A	0.5841 (4)	0.9068 (3)	0.2930 (2)	0.0778 (12)	
O5B	0.4012 (2)	0.5021 (2)	0.41180 (12)	0.0781 (8)	
O6B	0.4292 (2)	0.1793 (2)	0.24830 (12)	0.0734 (8)	
O7B	0.3404 (2)	0.7627 (2)	0.47626 (12)	0.0757 (8)	
O8B	0.2062 (2)	0.7323 (2)	0.33296 (11)	0.0615 (7)	
N3B	0.3194 (2)	0.3572 (2)	0.22398 (13)	0.0472 (7)	
N4B	0.2769 (2)	0.4843 (2)	0.26183 (13)	0.0474 (7)	
C13B	0.3189 (3)	0.5460 (3)	0.34960 (16)	0.0483 (9)	
C14B	0.3933 (4)	0.3432 (3)	0.37963 (18)	0.0704 (11)	
C15B	0.3850 (3)	0.2857 (3)	0.27812 (17)	0.0536 (9)	
C16B	0.2910 (3)	0.3093 (3)	0.12345 (15)	0.0423 (8)	
C17B	0.3345 (3)	0.4229 (3)	0.08030 (17)	0.0526 (9)	
C18B	0.3045 (3)	0.3784 (4)	−0.01600 (18)	0.0618 (11)	
C19B	0.2330 (3)	0.2206 (3)	−0.06714 (17)	0.0590 (10)	
C20B	0.1896 (3)	0.1073 (3)	−0.02371 (18)	0.0611 (10)	
C21B	0.2172 (3)	0.1515 (3)	0.07198 (17)	0.0546 (9)	
C22B	0.2899 (3)	0.6925 (3)	0.39472 (17)	0.0522 (9)	
C23B	0.1793 (3)	0.8789 (3)	0.36856 (19)	0.0650 (11)	
C24B	0.0808 (3)	0.8990 (3)	0.2889 (2)	0.0775 (12)	
H2A1	0.80160	0.27820	0.39260	0.0920*	
H2A2	0.98330	0.33580	0.41530	0.0920*	
H5A	0.56050	0.20300	0.10020	0.0630*	
H6A	0.51290	0.12080	−0.06110	0.0710*	
H7A	0.71450	0.18480	−0.13000	0.0700*	
H8A	0.96490	0.32670	−0.04020	0.0700*	
H9A	1.01380	0.41210	0.12100	0.0650*	
H11A	0.77990	0.97130	0.39730	0.0780*	
H11B	0.62810	0.87900	0.42190	0.0780*	
H12A	0.64050	0.91920	0.24650	0.1170*	
H12B	0.55990	0.99950	0.31520	0.1170*	
H12C	0.48950	0.81640	0.26630	0.1170*	
H14A	0.30240	0.27520	0.39190	0.0850*	
H14B	0.48420	0.33410	0.41520	0.0850*	
H17B	0.38380	0.52880	0.11560	0.0630*	
H18B	0.33210	0.45420	−0.04610	0.0740*	
H19B	0.21390	0.19040	−0.13190	0.0710*	
H20B	0.14180	0.00130	−0.05890	0.0730*	
H21B	0.18650	0.07580	0.10180	0.0660*	
H23A	0.27740	0.96760	0.39330	0.0780*	
H23B	0.12680	0.87300	0.41810	0.0780*	
H24A	0.13650	0.91110	0.24200	0.1160*	
H24B	0.05550	0.99110	0.31050	0.1160*	
H24C	−0.01310	0.80770	0.26270	0.1160*	

### Atomic displacement parameters (Å^2^)

**Table:**

	*U*^11^	*U*^22^	*U*^33^	*U*^12^	*U*^13^	*U*^23^
O1A	0.1398 (19)	0.0665 (13)	0.0441 (12)	0.0626 (14)	−0.0008 (11)	0.0092 (10)
O2A	0.149 (2)	0.0725 (14)	0.0581 (13)	0.0775 (15)	0.0200 (12)	0.0185 (11)
O3A	0.1091 (16)	0.0733 (14)	0.0425 (12)	0.0502 (13)	0.0084 (10)	0.0023 (10)
O4A	0.0855 (13)	0.0551 (12)	0.0523 (11)	0.0435 (11)	0.0094 (9)	0.0070 (9)
N1A	0.0726 (14)	0.0467 (12)	0.0389 (12)	0.0355 (11)	0.0130 (10)	0.0119 (10)
N2A	0.0628 (13)	0.0466 (13)	0.0433 (13)	0.0308 (11)	0.0118 (10)	0.0078 (10)
C1A	0.0688 (17)	0.0472 (16)	0.0420 (16)	0.0269 (14)	0.0091 (12)	0.0114 (13)
C2A	0.130 (3)	0.0616 (19)	0.0494 (18)	0.0566 (19)	0.0152 (16)	0.0158 (15)
C3A	0.089 (2)	0.0500 (17)	0.0475 (17)	0.0387 (16)	0.0135 (14)	0.0171 (14)
C4A	0.0540 (15)	0.0418 (14)	0.0389 (14)	0.0270 (12)	0.0097 (11)	0.0109 (11)
C5A	0.0491 (15)	0.0557 (17)	0.0525 (16)	0.0221 (13)	0.0155 (12)	0.0116 (13)
C6A	0.0495 (16)	0.0674 (18)	0.0533 (17)	0.0251 (14)	0.0037 (13)	0.0080 (14)
C7A	0.077 (2)	0.0672 (19)	0.0397 (15)	0.0435 (16)	0.0124 (14)	0.0117 (14)
C8A	0.0625 (17)	0.0724 (19)	0.0577 (18)	0.0366 (15)	0.0271 (14)	0.0264 (15)
C9A	0.0483 (15)	0.0568 (17)	0.0581 (17)	0.0235 (13)	0.0114 (12)	0.0156 (14)
C10A	0.0669 (17)	0.0499 (16)	0.0464 (16)	0.0282 (14)	0.0140 (13)	0.0096 (13)
C11A	0.0750 (19)	0.0528 (17)	0.0652 (19)	0.0331 (15)	0.0164 (15)	0.0033 (14)
C12A	0.098 (2)	0.078 (2)	0.077 (2)	0.0574 (19)	0.0208 (17)	0.0264 (18)
O5B	0.1242 (17)	0.0720 (14)	0.0437 (11)	0.0611 (13)	−0.0019 (10)	0.0102 (10)
O6B	0.1116 (16)	0.0736 (14)	0.0603 (13)	0.0652 (13)	0.0217 (11)	0.0245 (11)
O7B	0.1060 (16)	0.0771 (14)	0.0410 (11)	0.0505 (13)	0.0045 (10)	0.0002 (10)
O8B	0.0810 (13)	0.0600 (12)	0.0489 (11)	0.0438 (11)	0.0083 (9)	0.0084 (9)
N3B	0.0641 (13)	0.0467 (12)	0.0399 (12)	0.0330 (11)	0.0126 (10)	0.0134 (10)
N4B	0.0577 (13)	0.0451 (12)	0.0418 (12)	0.0283 (11)	0.0086 (9)	0.0077 (10)
C13B	0.0578 (16)	0.0470 (15)	0.0394 (15)	0.0233 (13)	0.0056 (12)	0.0110 (12)
C14B	0.111 (2)	0.0634 (19)	0.0514 (17)	0.0511 (18)	0.0157 (15)	0.0216 (15)
C15B	0.0681 (17)	0.0512 (16)	0.0485 (16)	0.0303 (14)	0.0134 (13)	0.0176 (14)
C16B	0.0453 (14)	0.0443 (15)	0.0402 (14)	0.0239 (12)	0.0109 (11)	0.0083 (12)
C17B	0.0541 (15)	0.0503 (16)	0.0542 (17)	0.0209 (13)	0.0102 (12)	0.0174 (14)
C18B	0.0666 (18)	0.081 (2)	0.0532 (17)	0.0378 (17)	0.0191 (14)	0.0312 (16)
C19B	0.0553 (17)	0.083 (2)	0.0397 (15)	0.0378 (16)	0.0083 (12)	0.0067 (16)
C20B	0.0577 (17)	0.0594 (18)	0.0550 (18)	0.0253 (15)	0.0054 (13)	−0.0013 (15)
C21B	0.0563 (16)	0.0498 (16)	0.0551 (17)	0.0231 (14)	0.0108 (12)	0.0089 (14)
C22B	0.0593 (16)	0.0550 (17)	0.0440 (16)	0.0280 (14)	0.0106 (12)	0.0105 (13)
C23B	0.078 (2)	0.0525 (17)	0.0672 (19)	0.0377 (15)	0.0175 (15)	0.0060 (14)
C24B	0.098 (2)	0.078 (2)	0.084 (2)	0.0613 (19)	0.0261 (18)	0.0335 (18)

### Geometric parameters (Å, º)

**Table:**

O1A—C1A	1.340 (3)	C5A—H5A	0.9300
O1A—C2A	1.427 (4)	C6A—H6A	0.9300
O2A—C3A	1.206 (4)	C7A—H7A	0.9300
O3A—C10A	1.199 (3)	C8A—H8A	0.9300
O4A—C10A	1.320 (3)	C9A—H9A	0.9300
O4A—C11A	1.462 (3)	C11A—H11B	0.9700
O5B—C13B	1.345 (3)	C11A—H11A	0.9700
O5B—C14B	1.423 (3)	C12A—H12A	0.9600
O6B—C15B	1.210 (3)	C12A—H12B	0.9600
O7B—C22B	1.195 (3)	C12A—H12C	0.9600
O8B—C22B	1.323 (3)	C13B—C22B	1.503 (4)
O8B—C23B	1.462 (3)	C14B—C15B	1.493 (4)
N1A—C4A	1.444 (3)	C16B—C17B	1.375 (4)
N1A—C3A	1.365 (3)	C16B—C21B	1.383 (4)
N1A—N2A	1.390 (3)	C17B—C18B	1.383 (4)
N2A—C1A	1.272 (3)	C18B—C19B	1.380 (4)
N3B—N4B	1.393 (3)	C19B—C20B	1.376 (4)
N3B—C16B	1.446 (3)	C20B—C21B	1.376 (4)
N3B—C15B	1.365 (3)	C23B—C24B	1.489 (4)
N4B—C13B	1.265 (3)	C14B—H14A	0.9700
C1A—C10A	1.503 (4)	C14B—H14B	0.9700
C2A—C3A	1.491 (4)	C17B—H17B	0.9300
C4A—C5A	1.382 (4)	C18B—H18B	0.9300
C4A—C9A	1.374 (4)	C19B—H19B	0.9300
C5A—C6A	1.380 (3)	C20B—H20B	0.9300
C6A—C7A	1.372 (4)	C21B—H21B	0.9300
C7A—C8A	1.374 (4)	C23B—H23A	0.9700
C8A—C9A	1.380 (3)	C23B—H23B	0.9700
C11A—C12A	1.488 (4)	C24B—H24A	0.9600
C2A—H2A1	0.9700	C24B—H24B	0.9600
C2A—H2A2	0.9700	C24B—H24C	0.9600
			
O1A···O3A	2.669 (3)	C10A···H23B^ix^	3.0100
O1A···N1A	2.701 (3)	C15B···H21B	2.8500
O1A···O1A^i^	3.032 (3)	C16B···H5A	3.0200
O1A···C2A^i^	3.150 (3)	C16B···H9A^vi^	3.0300
O2A···C9A	3.272 (3)	C18B···H8A^vi^	3.0100
O3A···C14B^ii^	3.418 (4)	C19B···H6A	3.0300
O3A···O1A	2.669 (3)	C19B···H8A^vi^	3.0200
O4A···N2A	2.644 (3)	C21B···H5A	3.0400
O5B···N3B	2.724 (3)	C22B···H11B	3.0100
O5B···O7B	2.667 (3)	C24B···H19B^x^	3.0800
O5B···O5B^ii^	3.024 (3)	H2A1···O7B^ii^	2.6200
O5B···C14B^ii^	3.163 (3)	H2A2···O3A^i^	2.6600
O6B···C21B	2.976 (3)	H2A2···O1A^i^	2.5500
O7B···O5B	2.667 (3)	H5A···O6B	2.7700
O8B···N4B	2.638 (3)	H5A···C16B	3.0200
O1A···H2A2^i^	2.5500	H5A···C21B	3.0400
O2A···H24B^iii^	2.7200	H6A···C5A^iv^	3.0900
O2A···H20B^iv^	2.8100	H6A···C19B	3.0300
O3A···H2A2^i^	2.6600	H7A···H12C^viii^	2.5800
O3A···H11B	2.6500	H7A···H24A^viii^	2.5400
O3A···H11A	2.6800	H8A···C18B^ix^	3.0100
O3A···H14A^ii^	2.5900	H8A···C19B^ix^	3.0200
O5B···H14B^ii^	2.5700	H9A···N4B^ix^	2.7700
O6B···H12B^v^	2.6600	H9A···C16B^ix^	3.0300
O6B···H5A	2.7700	H11A···O3A	2.6800
O6B···H21B	2.6500	H11B···C22B	3.0100
O7B···H11B	2.9100	H11B···O3A	2.6500
O7B···H23A	2.6900	H11B···O7B	2.9100
O7B···H2A1^ii^	2.6200	H12A···H19B^viii^	2.5900
O7B···H14B^ii^	2.6500	H12B···O6B^xi^	2.6600
O7B···H23B	2.6400	H12C···H7A^viii^	2.5800
N1A···O1A	2.701 (3)	H14A···O3A^ii^	2.5900
N2A···O4A	2.644 (3)	H14B···O5B^ii^	2.5700
N3B···O5B	2.724 (3)	H14B···O7B^ii^	2.6500
N4B···O8B	2.638 (3)	H17B···N4B	2.6600
N4B···H17B	2.6600	H18B···C5A^viii^	3.0400
N4B···H9A^vi^	2.7700	H19B···C24B^x^	3.0800
C2A···O1A^i^	3.150 (3)	H19B···H12A^viii^	2.5900
C7A···C21B^iv^	3.596 (4)	H19B···H24C^x^	2.5200
C8A···C8A^vii^	3.562 (4)	H20B···O2A^iv^	2.8100
C9A···O2A	3.272 (3)	H21B···O6B	2.6500
C14B···O3A^ii^	3.418 (4)	H21B···C15B	2.8500
C14B···O5B^ii^	3.163 (3)	H21B···C7A^iv^	3.0000
C18B···C18B^viii^	3.544 (5)	H23A···O7B	2.6900
C21B···O6B	2.976 (3)	H23B···O7B	2.6400
C21B···C7A^iv^	3.596 (4)	H23B···C10A^vi^	3.0100
C5A···H6A^iv^	3.0900	H24A···H7A^viii^	2.5400
C5A···H18B^viii^	3.0400	H24B···O2A^xii^	2.7200
C7A···H21B^iv^	3.0000	H24C···H19B^x^	2.5200
			
C1A—O1A—C2A	114.7 (2)	H12B—C12A—H12C	109.00
C10A—O4A—C11A	116.1 (2)	C11A—C12A—H12A	110.00
C13B—O5B—C14B	114.6 (2)	C11A—C12A—H12B	109.00
C22B—O8B—C23B	116.02 (19)	C11A—C12A—H12C	109.00
C3A—N1A—C4A	121.4 (2)	H12A—C12A—H12B	109.00
N2A—N1A—C3A	123.2 (2)	H12A—C12A—H12C	110.00
N2A—N1A—C4A	115.21 (19)	O5B—C13B—N4B	127.0 (2)
N1A—N2A—C1A	116.5 (2)	O5B—C13B—C22B	112.3 (2)
N4B—N3B—C16B	114.74 (19)	N4B—C13B—C22B	120.5 (2)
C15B—N3B—C16B	122.9 (2)	O5B—C14B—C15B	114.3 (2)
N4B—N3B—C15B	122.32 (19)	O6B—C15B—N3B	124.3 (2)
N3B—N4B—C13B	117.6 (2)	O6B—C15B—C14B	120.6 (2)
O1A—C1A—N2A	126.9 (3)	N3B—C15B—C14B	115.0 (2)
O1A—C1A—C10A	112.7 (2)	N3B—C16B—C17B	119.1 (2)
N2A—C1A—C10A	120.1 (2)	N3B—C16B—C21B	119.9 (2)
O1A—C2A—C3A	114.0 (2)	C17B—C16B—C21B	121.0 (2)
N1A—C3A—C2A	114.7 (2)	C16B—C17B—C18B	119.4 (3)
O2A—C3A—C2A	121.8 (3)	C17B—C18B—C19B	119.5 (3)
O2A—C3A—N1A	123.5 (2)	C18B—C19B—C20B	120.9 (2)
C5A—C4A—C9A	121.1 (2)	C19B—C20B—C21B	119.7 (3)
N1A—C4A—C5A	119.4 (2)	C16B—C21B—C20B	119.5 (2)
N1A—C4A—C9A	119.5 (2)	O7B—C22B—O8B	125.9 (3)
C4A—C5A—C6A	119.0 (3)	O7B—C22B—C13B	122.5 (2)
C5A—C6A—C7A	120.0 (3)	O8B—C22B—C13B	111.6 (2)
C6A—C7A—C8A	120.7 (2)	O8B—C23B—C24B	107.2 (2)
C7A—C8A—C9A	119.8 (3)	O5B—C14B—H14A	109.00
C4A—C9A—C8A	119.4 (3)	O5B—C14B—H14B	109.00
O3A—C10A—O4A	125.7 (3)	C15B—C14B—H14A	109.00
O3A—C10A—C1A	122.2 (3)	C15B—C14B—H14B	109.00
O4A—C10A—C1A	112.1 (2)	H14A—C14B—H14B	108.00
O4A—C11A—C12A	107.0 (2)	C16B—C17B—H17B	120.00
C3A—C2A—H2A1	109.00	C18B—C17B—H17B	120.00
C3A—C2A—H2A2	109.00	C17B—C18B—H18B	120.00
O1A—C2A—H2A2	109.00	C19B—C18B—H18B	120.00
O1A—C2A—H2A1	109.00	C18B—C19B—H19B	120.00
H2A1—C2A—H2A2	108.00	C20B—C19B—H19B	120.00
C6A—C5A—H5A	121.00	C19B—C20B—H20B	120.00
C4A—C5A—H5A	120.00	C21B—C20B—H20B	120.00
C5A—C6A—H6A	120.00	C16B—C21B—H21B	120.00
C7A—C6A—H6A	120.00	C20B—C21B—H21B	120.00
C8A—C7A—H7A	120.00	O8B—C23B—H23A	110.00
C6A—C7A—H7A	120.00	O8B—C23B—H23B	110.00
C9A—C8A—H8A	120.00	C24B—C23B—H23A	110.00
C7A—C8A—H8A	120.00	C24B—C23B—H23B	110.00
C4A—C9A—H9A	120.00	H23A—C23B—H23B	109.00
C8A—C9A—H9A	120.00	C23B—C24B—H24A	109.00
C12A—C11A—H11A	110.00	C23B—C24B—H24B	110.00
C12A—C11A—H11B	110.00	C23B—C24B—H24C	109.00
O4A—C11A—H11A	110.00	H24A—C24B—H24B	109.00
O4A—C11A—H11B	110.00	H24A—C24B—H24C	109.00
H11A—C11A—H11B	109.00	H24B—C24B—H24C	110.00
			
C2A—O1A—C1A—N2A	−24.5 (4)	N4B—N3B—C15B—O6B	−176.9 (2)
C2A—O1A—C1A—C10A	161.4 (3)	C15B—N3B—C16B—C21B	48.9 (4)
C1A—O1A—C2A—C3A	36.4 (4)	N3B—N4B—C13B—O5B	−0.1 (4)
C11A—O4A—C10A—O3A	3.7 (4)	N3B—N4B—C13B—C22B	174.4 (2)
C11A—O4A—C10A—C1A	−174.9 (2)	N2A—C1A—C10A—O3A	−172.7 (3)
C10A—O4A—C11A—C12A	−180.0 (3)	N2A—C1A—C10A—O4A	6.0 (4)
C13B—O5B—C14B—C15B	35.2 (4)	O1A—C1A—C10A—O4A	−179.4 (2)
C14B—O5B—C13B—N4B	−22.8 (4)	O1A—C1A—C10A—O3A	1.9 (4)
C14B—O5B—C13B—C22B	162.3 (2)	O1A—C2A—C3A—O2A	156.5 (3)
C22B—O8B—C23B—C24B	−178.7 (2)	O1A—C2A—C3A—N1A	−25.5 (4)
C23B—O8B—C22B—C13B	−176.4 (2)	N1A—C4A—C5A—C6A	179.9 (2)
C23B—O8B—C22B—O7B	2.4 (4)	C5A—C4A—C9A—C8A	−1.3 (4)
N2A—N1A—C3A—O2A	179.0 (3)	C9A—C4A—C5A—C6A	0.9 (4)
N2A—N1A—C3A—C2A	1.0 (4)	N1A—C4A—C9A—C8A	179.7 (2)
C4A—N1A—N2A—C1A	−171.4 (2)	C4A—C5A—C6A—C7A	−0.5 (4)
C3A—N1A—C4A—C9A	−78.9 (3)	C5A—C6A—C7A—C8A	0.4 (4)
N2A—N1A—C4A—C5A	−73.3 (3)	C6A—C7A—C8A—C9A	−0.8 (4)
C4A—N1A—C3A—O2A	4.0 (4)	C7A—C8A—C9A—C4A	1.2 (4)
C4A—N1A—C3A—C2A	−174.0 (2)	O5B—C13B—C22B—O7B	2.5 (4)
C3A—N1A—N2A—C1A	13.3 (3)	O5B—C13B—C22B—O8B	−178.7 (2)
N2A—N1A—C4A—C9A	105.7 (3)	N4B—C13B—C22B—O7B	−172.8 (3)
C3A—N1A—C4A—C5A	102.1 (3)	N4B—C13B—C22B—O8B	6.0 (4)
N1A—N2A—C1A—O1A	−1.0 (4)	O5B—C14B—C15B—O6B	155.0 (3)
N1A—N2A—C1A—C10A	172.8 (2)	O5B—C14B—C15B—N3B	−27.5 (4)
C15B—N3B—N4B—C13B	8.9 (3)	N3B—C16B—C17B—C18B	−178.5 (3)
C16B—N3B—N4B—C13B	−170.1 (2)	C21B—C16B—C17B—C18B	−0.3 (5)
N4B—N3B—C15B—C14B	5.8 (4)	N3B—C16B—C21B—C20B	179.5 (3)
C16B—N3B—C15B—O6B	2.0 (4)	C17B—C16B—C21B—C20B	1.3 (5)
C16B—N3B—C15B—C14B	−175.3 (2)	C16B—C17B—C18B—C19B	−0.7 (5)
N4B—N3B—C16B—C17B	46.1 (3)	C17B—C18B—C19B—C20B	0.8 (5)
N4B—N3B—C16B—C21B	−132.1 (3)	C18B—C19B—C20B—C21B	0.2 (5)
C15B—N3B—C16B—C17B	−132.8 (3)	C19B—C20B—C21B—C16B	−1.2 (4)

Symmetry codes: (i) −*x*+2, −*y*+1, −*z*+1; (ii) −*x*+1, −*y*+1, −*z*+1; (iii) *x*+1, *y*−1, *z*; (iv) −*x*+1, −*y*, −*z*; (v) *x*, *y*−1, *z*; (vi) *x*−1, *y*, *z*; (vii) −*x*+2, −*y*+1, −*z*; (viii) −*x*+1, −*y*+1, −*z*; (ix) *x*+1, *y*, *z*; (x) −*x*, −*y*+1, −*z*; (xi) *x*, *y*+1, *z*; (xii) *x*−1, *y*+1, *z*.

### Hydrogen-bond geometry (Å, º)

**Table:**

*D*—H···*A*	*D*—H	H···*A*	*D*···*A*	*D*—H···*A*
C2*A*—H2*A*2···O1*A*^i^	0.97	2.55	3.150 (3)	120
C14*B*—H14*A*···O3*A*^ii^	0.97	2.59	3.418 (4)	143
C14*B*—H14*B*···O5*B*^ii^	0.97	2.57	3.163 (3)	120

Symmetry codes: (i) −*x*+2, −*y*+1, −*z*+1; (ii) −*x*+1, −*y*+1, −*z*+1.
